# Multinational Survey on the Symptom Approach to Diagnosis and Therapy Adopted by Physicians in the Treatment of Gastrointestinal Sarcoidosis

**DOI:** 10.3390/jcm14228231

**Published:** 2025-11-20

**Authors:** Salvatore Nicolosi, Giorgio Monteleone, Giulia Bandini, Michael Hughes, Zsuzsanna McMahan, Elena Bargagli, Filippo Martone, Gianluca Ziosi, Paola Confalonieri, Marco Confalonieri, Paolo Cameli, Barbara Ruaro

**Affiliations:** 1 Pulmonology Unit, Department of Medical Surgical and Health Sciences, University Hospital of Cattinara, University of Trieste, 34149 Trieste, Italy; 2 Department of Cardiovascular and Pulmonary Sciences, Catholic University of Sacred Heart, 00168 Rome, Italy; 3 Department of Pneumology, Ruhrlandklinik, University Hospital, University of Duisburg-Essen, 45147 Essen, Germany; 4 Department of Experimental and Clinical Medicine, Division of Internal Medicine, Azienda Ospedaliera Universitaria Careggi, University of Florence, 50134 Florence, Italy; 5 Centre for Musculoskeletal Research, Division of Musculoskeletal and Dermatological Science, School of Biological Sciences, The University of Manchester, Manchester M13 9PT, UK; 6 Department of Rheumatology, Northern Care Alliance NHS Foundation Trust, The University of Manchester, Manchester M13 9PL, UK; 7 NIHR Manchester Biomedical Research Centre, Manchester University NHS Foundation Trust, Manchester M6 8HD, UK; 8 Division of Rheumatology, Department of Medicine, The University of Texas Health Science Center at Houston, Houston, TX 77030-5401, USA; 9 Respiratory Diseases Unit, Department of Medicine, Surgery and Neurosciences, University of Siena, 53100 Siena, Italy; 10 Amici Contro la Sarcoidosi Italia ets, 40137 Bologna, Italy; 11 Associazione Nazionale per l’Assistenza e la Ricerca Integrata Contro la Sarcoidosi, 00152 Roma, Italy

**Keywords:** gastrointestinal sarcoidosis, glucocorticoids, biological therapies, multidisciplinary approach

## Abstract

**Background:** Gastrointestinal (GI) sarcoidosis is a rare manifestation of systemic disease, with limited evidence to guide diagnosis and treatment. **Methods:** A 75-item international survey was completed by 132 clinicians from multiple specialties and hospital settings. Statistical analysis was performed using Jamovi version 2.6.44 (The Jamovi Project, Sydney, Australia). **Results:** Most clinicians (72.0%) preferred a comprehensive diagnostic approach integrating clinical, imaging and histopathological assessment, with differences by hospital type (*p* < 0.05). Inflammatory bowel disease was frequently considered in the differential diagnosis, and concern regarding excluding inflammatory conditions remained consistently high. Significant specialty-related variation was observed for specific organ involvement and in the management of glucocorticoid-refractory disease, including use of alternative immunosuppressants, combination regimens, glucocorticoid-sparing agents, and biologic therapies. Expectations regarding response timelines and indications for surgery were largely concordant. **Conclusions:** Clinicians reported high awareness but heterogeneous management practices for GI sarcoidosis, particularly beyond first-line glucocorticoids. These findings highlight the need for multidisciplinary consensus and the development of standardized clinical guidelines.

## 1. Introduction

Sarcoidosis is a chronic, systemic granulomatous disease characterized by a heterogeneous clinical presentation and a variable, often unpredictable course [1,2]. Although its pathogenesis remains unclear, the most accredited hypothesis is represented by the interplay between genetic predisposition (polymorphisms and gene variants) and environmental factors, including microbial infections (e.g., Mycobacterium tuberculosis, Propionibacterium acnes), metal and organic dust, silica and inhaled antigens [1,2,3]. This interaction led to an exaggerated immune response which results in the formation of granulomatous lesions that can be located in the lungs and hilar/mediastinal lymph nodes or in extrapulmonary sites [1,2,3,4,5].

Although the lung involvement occurs in approximately 90% of patients, it can virtually affect every organ; notably, demographics (i.e., sex, age at presentation, ethnicity) and genetic background may influence clinical presentation across organ systems, including disease course and prognosis [6,7]. Amid the extrapulmonary manifestations of sarcoidosis, gastrointestinal involvement (GI) is a rare occurrence, with reported incidence of less than 1%, excluding hepatic and splenic involvement [2,3]. It typically occurs in middle-aged patients with systemic disease, and GI symptoms are reported in 0.1% to 0.9% of cases [8,9,10,11,12,13]. Within the GI tract, the stomach is the most commonly affected site, whereas involvement of the esophagus and small bowel is extremely rare [13,14,15,16,17]. In addition, the pancreas and large intestine can be additional localizations of GI sarcoidosis, which may mimic inflammatory diseases (e.g., pancreatitis, colitis) or malignancies due to their non-specific symptoms [18,19,20]. However, GI sarcoidosis is uncommon, and its diagnosis relies on a combination of imaging, endoscopy, and histopathology [5,21,22,23]. Due to the heterogeneous origin of granulomas, more common causes of granuloma formation such as infections (e.g., tuberculosis, non-tuberculous mycobacteria and Whipple’s disease), inflammatory disorders (e.g., Crohn’s disease), immunodeficiency, and sarcoid-like reactions induced by cancers or medications, should be carefully excluded [21,22,23]. Hence, this survey aimed at exploring clinicians’ diagnostic and therapeutic approaches to GI sarcoidosis, with a focus on awareness of differential diagnoses, use of glucocorticoid-sparing and biologic therapies, expectations regarding treatment response timelines, and variation in management across specialties and hospital types. By synthesizing these perspectives, the study provides a foundation for developing standardized recommendations and consensus guidelines for the management of this rare condition.

## 2. Materials and Methods

### 2.1. Study Design and Objectives

A cross-sectional survey study was conducted to investigate clinicians’ perspectives and clinical approaches to GI involvement in sarcoidosis. The primary objective was to explore awareness, diagnostic strategies, and therapeutic preferences among clinicians managing patients with sarcoidosis, with a particular focus on GI manifestations, which remain rare and underrecognized.

### 2.2. Survey Development

The survey was developed by a multidisciplinary team of specialists in internal medicine, gastroenterology, and respiratory medicine. It comprised 75 items organized into thematic sections, covering clinician demographics and professional background (i.e., age, sex, country, specialty, hospital type, years in practice, volume of sarcoidosis patients managed annually); perceptions of clinical presentation and diagnostic strategies for GI sarcoidosis; and therapeutic approaches, including the use of glucocorticoids, immunosuppressants, and biologics, as well as expectations regarding treatment response (Supplementary Material S1). Both single-choice and multiple-response questions were included. The preliminary version was pilot-tested with ten clinicians, after which minor revisions were made to improve clarity and content validity.

### 2.3. Data Collection and Clinicians Recruitment

The final version of the survey was distributed electronically between November 2024 and April 2025 via a web-based form, accessible through a direct link or QR code. Recruitment occurred through professional societies, specialty mailing lists, and conferences related to sarcoidosis. In addition, some clinicians were approached personally and invited to participate using the same electronic survey link. Participation was voluntary and anonymous. No personal or identifying data were collected, ensuring that respondents could not be individually traced. Anonymity was assured for all participants, including those recruited through personal contact.

### 2.4. Inclusion Criteria

Eligible participants were physicians of any specialty involved in the management of patients with sarcoidosis. No geographic or institutional restrictions were applied. Questionnaires with major missing sections were excluded from the analysis.

### 2.5. Data Handling and Ethics

Data were handled in accordance with ethical standards for anonymous survey research and the principles of the Declaration of Helsinki. The study was approved by the Institutional Ethics Committee of the University of Trieste (protocol 2024-9, 29 October 2024). No patient data, clinical interventions, or sensitive personal identifiers were collected, and participant anonymity was maintained throughout. The study dataset is in line with the requirements of the Ethics Committee and institutional regulations, and may not be reused or shared without prior authorization from the Ethics Committee and the research group.

### 2.6. Statistical Analysis

All analyses were conducted using Jamovi version 2.6.44 (The Jamovi Project, Sydney, Australia). Descriptive statistics were reported as absolute frequencies and percentages. Chi-square (χ^2^) tests of independence assessed associations between categorical variables, and Kruskal–Wallis tests compared distributions of ordinal data, with effect sizes reported. Moreover, specialties with few respondents (immunologists, general surgeons, gastroenterological surgeons, general practitioners, and specialist nurses) were grouped as “other specialties.” A two-tailed significance level of *p* < 0.05 was applied, and missing data were handled by pairwise deletion.

## 3. Results

### 3.1. Demographics Analysis

A total of 222 clinicians replied to the survey, and 132 clinicians (59.5%) provided complete responses. Respondents represented a broad spectrum of professional backgrounds and healthcare settings. The majority were female (58.3%), while 41.7% were male. Most participants were aged 31–50 years (57.6%), reflecting a predominantly mid-career cohort; 25.0% were aged 18–30 years, and 17.4% were 51–70 years.

Respondents were primarily based in Italy (89.4%), with smaller proportions from the Netherlands (6.1%), the United Kingdom (1.5%), Belgium (0.8%), Denmark (0.8%), Germany (0.8%), and Ireland (0.8%). Most clinicians (74.2%) worked in general (non-university) teaching hospitals, while 25.8% were affiliated with university hospitals, indicating strong representation from Southern European non-academic centers (Table 1). The moderate completion rate suggests satisfactory engagement and adequate representativeness for exploratory analysis of clinicians’ perceptions and practices.

With regard to clinical experience, the number of patients with sarcoidosis managed by respondents varied considerably. Most clinicians (60.6%) reported following fewer than 25 patients with sarcoidosis, while 8.3% managed 25–30 patients, 9.1% managed 31–50, and 7.6% reported 51–100 patients. A smaller but notable group (14.4%) reported overseeing more than 100 patients, indicating the inclusion of both low- and high-volume centers.

When focusing on GI sarcoidosis, the majority of respondents (88.6%) indicated that GI involvement was observed in 5–10% of the patients. A further 9.8% of respondents reported 10–30% of cases with GI involvement, while only 1.6% reported higher proportions (≥30%), underscoring the perceived rarity of GI manifestations within the broader sarcoidosis population.

### 3.2. Perceptions, Clinical Manifestations, and Multidisciplinary Practices

**Figure 1:**
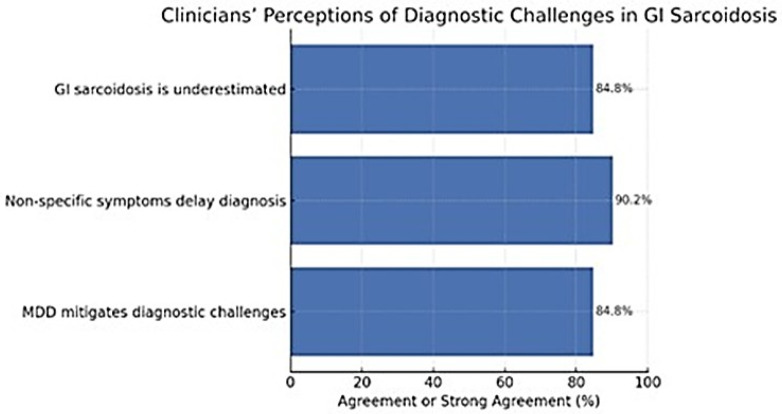
Clinicians’ perceptions of diagnostic challenges in gastrointestinal sarcoidosis. The majority agreed that the condition is often underestimated, that non-specific symptoms contribute to diagnostic delay, and that MDD helps mitigate these challenges. Abbreviations: GI, gastrointestinal; MDD, multidisciplinary discussion.

Clinicians reported a shared recognition of the diagnostic complexity associated with GI sarcoidosis. A large majority (84.8%) agreed or strongly agreed that the condition is often underestimated in clinical practice, and 90.2% believed that non-specific symptoms substantially contribute to diagnostic delays. Furthermore, 84.8% agreed that multidisciplinary discussion (MDD) can help mitigate these challenges, underscoring the perceived importance of collaborative evaluation. Agreement that MDD reduces diagnostic delay did not differ significantly across specialties (χ^2^ = 8.33; df = 9; *p* = 0.50; Cramér’s *V* = 0.15) or by hospital type (χ^2^ = 1.71; df = 3; *p* = 0.64; Cramér’s *V* = 0.11), indicating broad consensus across healthcare settings on the value of multidisciplinary care (Figure 1).

Reported GI symptoms were heterogeneous. Abdominal pain (56.1%) and dyspepsia (44.7%) were the most frequent manifestations, followed by unintentional weight loss (42.4%) and gastroesophageal reflux (37.9%). Abdominal distension or bloating was also common (31.1%), whereas dysphagia (23.5%) and diarrhea (17.4%) were less frequent. Early satiety (12.9%), constipation (12.1%), and upper gastrointestinal bleeding (3.8%) were comparatively rare. These findings highlight the non-specific nature of GI involvement, which may hinder timely recognition.

Referral and multidisciplinary involvement varied widely among respondents. Approximately one-third of clinicians (34.1%) reported referring patients often, and 21.2% always referred; others referred sometimes (22.7%), rarely (13.6%), or never (8.3%). The frequency of GI specialist consultation differed only marginally across specialties (χ^2^ = 19.9; df =12; *p* = 0.069; Cramér’s *V* = 0.22) and did not vary significantly by hospital type (χ^2^ = 2.68; df = 4; *p* = 0.61; Cramér’s *V* = 0.14). Overall, these findings suggest that while awareness of diagnostic challenges is high and the perceived value of multidisciplinary evaluation is widely acknowledged, its consistent implementation across specialties and healthcare settings remains suboptimal.

### 3.3. Diagnostic Concerns and Differential Diagnosis

Clinicians expressed high concern for excluding inflammatory (94.7%), infectious (88.6%), and malignant (86.4%) conditions when evaluating suspected GI sarcoidosis. Autoimmune diseases (82.6%) and structural abnormalities (79.5%) were also frequently considered.

Across specialties, Kruskal–Wallis tests revealed no significant differences in concern for inflammatory (H = 4.83; df = 3; *p* = 0.18; ε^2^ = 0.01), infectious (H = 5.80; df = 3; *p* = 0.12; ε^2^ = 0.02), malignant (H = 4.18; df = 3; *p* = 0.24; ε^2^ = 0.01), or autoimmune conditions (H = 2.50; df = 3; *p* = 0.48; ε^2^ = 0.00). However, concern for structural abnormalities varied significantly between groups (H = 9.64; df = 3; *p* = 0.022; ε^2^ = 0.05).

Consideration of inflammatory bowel disease (IBD) in the differential diagnosis of GI sarcoidosis did not differ significantly across specialties (χ^2^ = 12.4; df = 12; *p* = 0.41; Cramér’s *V* = 0.22) or hospital types (χ^2^ = 2.19; df = 4; *p* = 0.70; Cramér’s *V* = 0.09). Most clinicians reported evaluating IBD frequently, with 35.6% selecting “often” and 13.0% “always”. The proportion reporting “often” or “always” was slightly higher in university hospitals (34.9%) than in non-university centers (13.6%), suggesting a trend toward more systematic assessment in tertiary settings. Across specialties, rheumatologists (58.8%) and pneumologists (33.4%) most frequently considered IBD in the differential, followed by gastroenterologists (28.6%) and other specialists (46.2%).

Regarding sarcoid-like reactions to infliximab in Crohn’s disease, 60.6% of respondents reported no prior experience, while 37.1% indicated that such events always led to diagnostic reassessment. No significant differences were observed by specialty (χ^2^ = 8.83; df = 9; *p* = 0.45; Cramér’s *V* = 0.18) or hospital type (χ^2^ = 1.52; df = 3; *p* = 0.68; Cramér’s *V* = 0.11). Experience with such reactions was slightly more common among university hospital clinicians (27.3%) than among those in non-university centers (18.2%). Hence, these findings indicate limited but clinically relevant awareness of infliximab-related sarcoid-like reactions and emphasize the need for ongoing diagnostic vigilance when evaluating granulomatous findings in patients receiving anti-tumor necrosis factor therapy.

### 3.4. Diagnostic Tools and Practices

**Figure 2:**
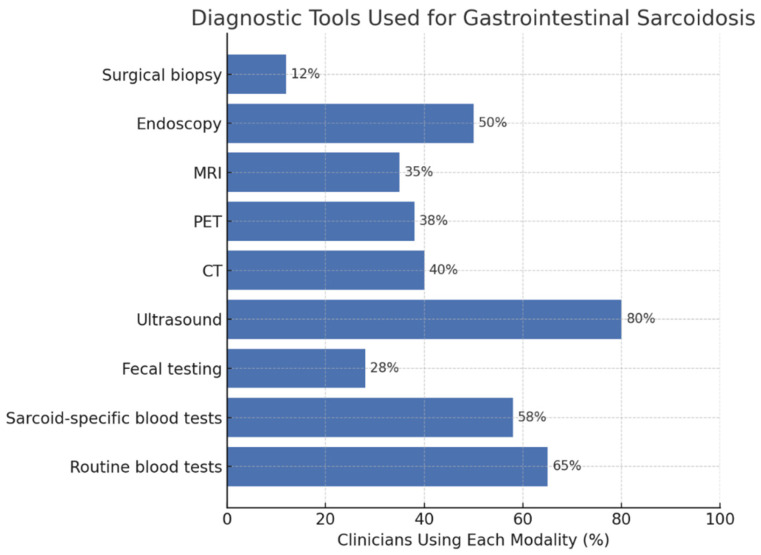
Diagnostic tools used for gastrointestinal sarcoidosis. Percentages indicate the proportion of clinicians reporting personal use of each diagnostic modality (N = 132). Abbreviations: MRI, magnetic resonance imaging; PET, positron emission tomography; CT, computed tomography.

Clinicians reported using a wide range of diagnostic modalities for evaluating suspected GI sarcoidosis (Figure 2). Routine blood testing (63.6%) and sarcoid-specific blood tests (56.8%) were the most frequently employed, while fecal testing was less common (28.8%). Among imaging modalities, ultrasound was most frequently used (80.3%), followed by computed tomography (38.6%), positron emission tomography (36.4%), and magnetic resonance imaging (32.6%). Additionally, endoscopy was reported by 47.7% of respondents, and surgical biopsy by 10.6%.

When asked which diagnostic tools they personally use, the choice of surgical biopsy differed significantly across specialties (χ^2^ = 12.4; df = 3; *p* = 0.006; Cramér’s *V* = 0.31) but not by hospital type (χ^2^ = 0.56; df =1; *p* = 0.45; Cramér’s *V* = 0.07). Specifically, surgical biopsy was selected by 29.4% of rheumatologists and 30.8% of clinicians grouped as “other specialties,” compared with 8.0% of pneumologists and none of the gastroenterologists. Biopsy use was slightly higher in university hospitals (13.4%) than in non-university centers (8.6%), though this difference was not statistically significant.

Similarly, the use of endoscopy did not vary significantly by hospital type (χ^2^ = 0.22; df =1; *p* = 0.64; Cramér’s *V* = 0.04) but differed significantly across specialties (χ^2^ = 8.49; df = 3; *p* = 0.037; Cramér’s *V* = 0.25). Gastroenterologists universally reported performing endoscopy (100%), whereas pneumologists were less likely to do so (65.9%). These findings indicate that procedural heterogeneity is primarily driven by specialty-related roles and expertise, whereas diagnostic practices remain broadly consistent across hospital types.

### 3.5. Experience and Diagnostic Reasoning in Clinicians Managing GI Sarcoidosis

Across specialties, concern regarding coexisting organ involvement varied significantly for several manifestations. Kruskal–Wallis tests showed significant differences for skin (H = 13.00; df = 3; *p* = 0.005; ε^2^ = 0.10), ocular (H = 14.75; df = 3; *p* = 0.002; ε^2^ = 0.11), parotid gland (H = 12.92; df = 3; *p* = 0.005; ε^2^ = 0.10), peripheral arthritis (H = 9.92; df = 3; *p* = 0.019; ε^2^ = 0.08), renal (H = 10.17; df = 3; *p* = 0.017; ε^2^ = 0.08), neurological (H = 9.73; df = 3; *p* = 0.021; ε^2^ = 0.07), pancreatic (H = 15.55; df = 3; *p* = 0.001; ε^2^ = 0.12), and rhino-laryngeal (H = 7.85; df = 3; *p* = 0.049; ε^2^ = 0.06) involvement. No significant differences were observed for pulmonary (H = 2.60; df = 3; *p* = 0.46; ε^2^ = 0.02), lymphatic (H = 3.42; df = 3; *p* = 0.33; ε^2^ = 0.03), splenic (H = 5.25; df = 3; *p* = 0.15; ε^2^ = 0.04), hepatic (H = 4.01; df = 3; *p* = 0.26; ε^2^ = 0.03), or cardiac (H = 5.80; df =3; *p* = 0.12; ε^2^ = 0.04) involvement. Generally, these findings indicate that clinicians’ attention to extrapulmonary manifestations is uneven, with higher vigilance toward skin, ocular, pancreatic, and parotid involvement, and comparatively lower concern for hepatic, splenic, and cardiac disease. When comparing clinicians from university versus non-university hospitals, Kruskal–Wallis analyses showed significant differences in prioritization only for pulmonary (H = 4.43; df = 1; *p* = 0.035; ε^2^ = 0.03) and borderline for neurological involvement (H = 3.86; df = 1; *p* = 0.050; ε^2^ = 0.03). No significant differences were found for lymphatic (H = 3.04; df = 1; *p* = 0.081), ocular (H = 2.85; df = 1; *p* = 0.091), pancreatic (H = 2.05; df = 1; *p* = 0.152), or cardiac (H = 2.59; df = 1; *p* = 0.108) involvement, nor for other systemic sites (all *p* > 0.20). These results suggest that clinicians in university hospitals may place slightly greater emphasis on pulmonary and neurological comorbidities when managing GI sarcoidosis, while overall patterns of concern remain largely consistent across institutional settings.

Consideration of IBD as a differential diagnosis did not vary significantly across specialties (χ^2^ = 12.4; df = 12; *p* = 0.41; Cramér’s *V* = 0.28). Most clinicians reported evaluating IBD frequently, with 35.6% selecting “often” and 13.0% “always.” Among specialties, gastroenterologists most frequently reported considering IBD (“rarely” 42.9%, “sometimes” 28.6%, “often” 14.3%, “always” 14.3%), followed by rheumatologists (“sometimes” 23.5%, “often” 29.4%, “always” 29.4%), pneumologists (“rarely” 23.9%, “sometimes” 23.9%, “often” 38.6%, “always” 11.4%), and clinicians in other specialties (“rarely” 38.5%, “sometimes” 15.4%, “often” 46.2%).

### 3.6. Glucocorticoid Use and Use of GI Protective Therapy

When clinicians were asked whether systemic therapy should be considered first-line treatment for isolated GI sarcoidosis, no significant differences emerged among specialties (χ^2^ = 3.06; df = 6; *p* = 0.80; Cramér’s *V* = 0.11) or hospital types (χ^2^ = 0.58; *p* = 0.75; Cramér’s *V* = 0.07); 68.9% of respondents agreed that systemic therapy particularly glucocorticoids (GCs) should represent the initial approach, while 5.3% disagreed and 25.8% were uncertain. Agreement was higher among rheumatologists (82.4%) and pneumologists (67.0%), and slightly lower among gastroenterologists (71.4%) and other specialists (61.5%). Most clinicians (79.5%) also agreed that therapeutic decisions should depend on the extent of extraintestinal involvement and the patient’s overall systemic condition, again without significant variation across specialties (χ^2^) = 11.4; df = 9; *p* = 0.25; Cramér’s *V* = 0.21) or hospital types (χ^2^ = 3.27; df = 3; *p* = 0.35; Cramér’s *V* = 0.16). Similarly, 68.2% indicated that therapy should vary according to the GI site affected, with comparable consensus across specialties (χ^2^ = 5.63; df = 12; *p* = 0.93; Cramér’s *V* = 0.17) and hospital types (χ^2^ = 3.57; df = 4; *p* = 0.47; Cramér’s *V* = 0.17). These findings highlight a strong overall consensus that management should be individualized according to systemic disease burden and anatomical site, with glucocorticoids (GCs) remaining the therapeutic cornerstone across specialties and institutions. Expected response times to high-dose GCs varied, with most anticipating improvement within one to two months (47.3%), followed by three to five months (24.0%) or less than one month (22.5%). These expectations did not differ by specialty (χ^2^ = 10.4; df = 9; *p* = 0.32; Cramér’s *V* = 0.16). Conversely, co-prescription of GI protective therapy varied significantly (χ^2^ = 25.1; df = 12; *p* = 0.014; Cramér’s *V* = 0.25): rheumatologists (88.2%) and pneumologists (77.3%) reported using protection “often” or “always” more frequently than gastroenterologists (50.0%) and other specialists (84.6%).

### 3.7. Post-Glucocorticoid Failure Strategies

After failure of maximal-dose GC therapy, most clinicians (82.6%) favored combination treatment, typically GC plus methotrexate, while smaller proportions opted to continue GC alone (9.1%), taper with dietary modification (15.9%), discontinue GC in favor of another immunosuppressant (24.2%), or consider invasive management (4.5%). Combination therapy was the predominant approach across specialties, endorsed by 100% of rheumatologists, 80.7% of pneumologists, 78.6% of gastroenterologists, and 76.9% of other specialists. Its use was comparably frequent among university (83.5%) and non-university clinicians (80.0%) (χ^2^ = 7.38; df = 6; *p* = 0.29). Discontinuation of GC in favor of another immunosuppressant was reported by 26.8% of university and 20.0% of non-university clinicians (χ^2^ = 8.75; df = 6; *p* = 0.19), while continuation of GC (6.8% vs. 8.6%), tapering with dietary modification (17.5% vs. 11.4%), and consideration of invasive management (6.2% vs. 0.0%) showed comparable distributions between hospital types (all *p* > 0.05).

In contrast, significant inter-specialty variation emerged in the use of steroid-sparing disease-modifying antirheumatic drugs (DMARDs) (χ^2^ = 35.1; df = 12; *p* < 0.001; Cramér’s *V* = 0.30). Rheumatologists were the most frequent prescribers (76.5% reporting “often” or “always”), followed by pneumologists (56.8%), gastroenterologists (35.7%), and other specialists (46.2%). Expectations regarding treatment response were consistent across specialties (χ^2^ = 9.18; df = 9; *p* = 0.42), with most anticipating clinical improvement within one to five months. Specifically, 47.3% of clinicians expected a response to high-dose GC therapy within one to two months, 24.0% within three to five months, 6.3% after approximately six months, and 22.5% within the first month. These expectations were comparable between university (32.6%, 17.8%, 4.7%, and 18.6%) and non-university clinicians (14.7%, 6.2%, 1.6%, and 3.9%), with no significant differences by hospital type (χ^2^ = 8.92; df = 6; *p* = 0.18; Cramér’s *V* = 0.20).

All in all, these findings reinforce the central role of GCs and combination regimens after GC failure, while highlighting inter-specialty differences in DMARD use and a shared recognition of both the gradual therapeutic onset of steroid-sparing agents and expected time to response with GC therapy in GI sarcoidosis.

### 3.8. Biologic Therapies and Emerging Treatments

Clinicians reported heterogeneous practices regarding biologic therapy for GC-refractory GI sarcoidosis. Overall, 37.1% used biologics “sometimes,” 31.1% “often,” and 6.1% “always,” while 18.9% used them “rarely” and 6.8% “never.” Usage did not differ significantly by specialty (χ^2^ = 17.9; df = 12; *p* = 0.12) or hospital type (χ^2^ = 2.64; df = 4; *p* = 0.62; Cramér’s *V* = 0.12), though clinicians in university hospitals reported slightly greater use (29.5% “often” or “always”) than those in non-university centers (21.6%). Biologics were most frequently prescribed by rheumatologists (70.6%) and pneumologists (55.7%), followed by gastroenterologists (35.7%) and other specialists (38.5%).

**Figure 3:**
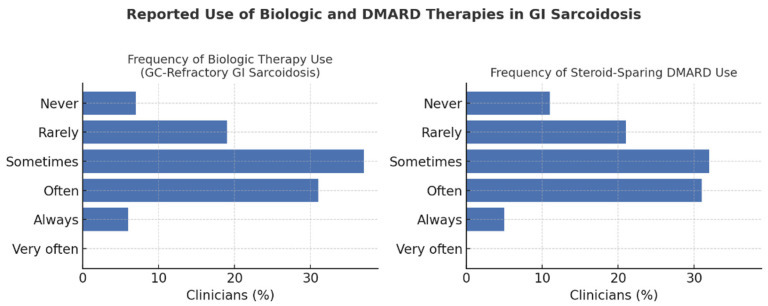
Reported use of biologic and steroid-sparing DMARD therapies in GI sarcoidosis. Bars indicate the percentage of clinicians reporting each frequency of use for biologics (**left**) and DMARDs (**right**) (N = 132). Abbreviations: DMARD, disease-modifying anti-rheumatic drugs; GI, gastrointestinal.

In contrast, DMARD use varied significantly by specialty (χ^2^ = 35.1; df = 12; *p* < 0.001), being most common among rheumatologists (76.5%), pneumologists (56.8%), gastroenterologists (35.7%), and other specialties (46.2%). Across respondents, 31.8% prescribed DMARDs “sometimes,” 31.1% “often,” and 5.3% “very often,” while 21.2% used them “rarely” and 10.6% “never” (Figure 3).

Most clinicians (82.6%) favored combination therapy—typically GC plus methotrexate—after GC failure, with no significant inter-specialty differences (χ^2^ = 4.25; df = 3; *p* = 0.24). Expected response times to biologics were also comparable, with 50.4% anticipating improvement within one to two months and 36.4% within three to five months.

Overall, these findings underscore glucocorticoids as the therapeutic backbone, with variable adoption of DMARDs and biologics but broadly consistent management attitudes across specialties and hospital settings.

### 3.9. Surgical Perspectives and Indications

Attitudes toward GI surgery were generally conservative across specialties and hospital settings. Only 15.5% of respondents agreed or strongly agreed that surgery should be pursued regardless of GC efficacy, whereas 39.4% remained neutral and 45.1% disagreed or strongly disagreed. No significant differences were observed by hospital type (χ^2^ = 1.18; df = 3; *p* = 0.76; Cramér’s *V* = 0.09) or specialty (χ^2^ = 8.21; df = 9; *p* = 0.51; Cramér’s *V* = 0.13). Disagreement was most frequent among pneumologists (71.0%) and gastroenterologists (76.9%), while rheumatologists (29.4%) and other specialists (29.4%) expressed slightly higher agreement. Perceptions of surgical efficacy varied modestly by anatomical site but remained consistent across hospital types (all *p* > 0.20) and specialties (*p*-values 0.43–0.51). Agreement that surgery could be effective was highest for large-bowel (42.6%) and anorectal (37.9%) involvement, intermediate for small bowel (32.6%) and stomach (29.5%), and lowest for the esophagus (28.6%) and oropharynx (27.9%). Hence, these results highlight a broad consensus that GI surgery should be reserved for refractory or anatomically complex disease, reflecting a shared preference for medical or combination therapy as the cornerstone of gastrointestinal sarcoidosis management.

## 4. Discussion

This multinational clinician survey provides an overview of current diagnostic and therapeutic practices for GI sarcoidosis across multiple specialties and healthcare settings. The findings reveal both convergence and divergence in clinical reasoning and management. Most clinicians emphasized the importance of excluding inflammatory, infectious, and malignant mimics and endorsed a multimodal diagnostic strategy integrating clinical, imaging, and histopathological data. Ultrasound and blood-based tests were the most frequently used modalities, whereas surgical biopsy was uncommon [24,25,26,27,28]. GC remained the preferred first-line treatment, and combination therapy was the dominant approach following GC failure. However, significant inter-specialty variation emerged in the use of steroid-sparing DMARDs and in the co-prescription of GI protective therapy, underscoring the absence of standardized guidance and the need for harmonized care pathways [2,26,27,28,29,30].

Sarcoidosis presents with diverse extrapulmonary manifestations that complicate diagnosis and management. Cutaneous involvement is the most common initial presentation (~30%), ocular disease develops later in 10–50% of cases, and cardiac sarcoidosis is found in 20–25% at autopsy, though only 5–10% are symptomatic. Musculoskeletal disease may be acute and self-limiting or chronic, involving joints or bone. Neurological involvement affects 5–10%, with neurosarcoidosis more frequent in both African and American patients, while non-granulomatous forms such as small-fiber neuropathy remain poorly defined but may also affect the GI system, particularly through sensory alterations and dysautonomic manifestations such as diarrhea, constipation, and dysmotility syndromes involving the esophagus, stomach, or intestines [5,24,25,26,27,28,29,30]. Even though these manifestations remain poorly characterized and underreported, their recognition may help identify subtle or early sarcoid involvement with distinct therapeutic implications [3]. Focusing specifically on the GI tract, sarcoid involvement appears to be much rarer and remains underrepresented in the literature [3,8,10,27,28,29,30,31,32,33].

Findings from our survey reveal substantial variability in diagnostic and therapeutic practices for GI sarcoidosis, highlighting the need for structured, evidence-based guidance. Standardized pathways could help distinguish GI sarcoidosis from mimicking conditions such as IBD, ensure timely biopsy and imaging, and clarify treatment sequencing in glucocorticoid-refractory cases [34,35,36,37,38,39,40,41,42,43]. Embedding MDD into care pathways may further harmonize decision-making and reduce diagnostic delay. Although tools such as ACCESS and STAI have been proposed to assess organ involvement and disease activity, specific diagnostic guidelines for extrapulmonary sarcoidosis remain lacking [35,36,37,43]. Therefore, our findings can serve as a foundation for future consensus statements and guidelines aimed at improving consistency and patient outcomes.

From 132 clinicians across multiple specialties and hospital settings, we identified both convergences and divergences in practice. A strong and consistent emphasis was placed on ruling out inflammatory conditions such as IBD, reflecting broad awareness across specialties of the diagnostic overlap between granulomatous disorders. Although inter-specialty differences were not statistically significant, the uniformly high concern underscores clinicians’ shared vigilance toward inflammatory mimics of GI sarcoidosis. Specialty-related differences emerged for skin, ocular, pancreatic, and parotid involvement, suggesting that clinical background shapes diagnostic priorities. Marked heterogeneity was also evident in treatment strategies for GC-refractory disease. Combination therapy with immunosuppressants was most widely endorsed, but significant differences across specialties were seen in continuing GCs, applying lifestyle and dietary measures, using alternative immunosuppressants, and adopting biologics.

This variation underscores the absence of unified guidance and the risk of inconsistent care. However, our findings point to the need to distinguish appropriate variability driven by patient-specific or contextual factors from unwarranted variability, which reflects knowledge gaps or lack of standards. Harmonization is most urgently required for diagnostic pathways (clinical, imaging, histology), therapeutic sequencing (steroids, immunosuppressants, biologics), and surgical referral criteria. Central to this is the role of MDD, which can reduce diagnostic delays, align interpretations, and support consistent treatment across specialties [35,36,37,38,39,40,41,42,43]. Therefore, this study represents one of the largest clinician-based surveys focused on GI sarcoidosis, with broad representation across specialties and institutions. Its strengths include a multidisciplinary questionnaire design, anonymous participation to minimize reporting bias, and systematic statistical analysis to enhance the reliability of the findings.

Nevertheless, several limitations should be acknowledged. Although 132 clinicians participated, the sample was geographically imbalanced, with most respondents based in Italy (89.4%) and only a small proportion from other European countries such as the Netherlands, the United Kingdom, Belgium, Denmark, Germany, and Ireland. This limited international representation may restrict the generalizability of the findings. The professional composition was also uneven, as pneumologists were the most represented specialty, while gastroenterologists and rheumatologists were comparatively fewer, potentially influencing inter-specialty comparisons. Moreover, most participants were employed in general (non-university) teaching hospitals (74.2%), potentially underrepresenting tertiary or research-oriented centers where complex sarcoidosis cases are more often managed. Additionally, most respondents reported caring for fewer than 25 sarcoidosis patients annually, reflecting limited individual experience with this rare condition. Because the survey relied on self-reported rather than observed clinical practices, recall and social desirability biases cannot be excluded. The cross-sectional design captures practices at a single time point and therefore cannot establish causal or temporal relationships. Finally, the survey did not explore how clinicians incorporate patients’ quality of life or preferences into clinical decision-making.

Despite these limitations, the present study provides valuable insights into diagnostic and therapeutic heterogeneity across specialties and institutions. It highlights key areas where standardization and consensus-based guidelines could enhance the consistency and quality of care for patients with GI sarcoidosis.

## 5. Conclusions

All in all, although awareness of GI sarcoidosis and its differential diagnoses is high, diagnostic and therapeutic strategies remain heterogeneous across specialties. These findings underscore the need for multidisciplinary consensus and standardized guidelines to optimize care for patients with GI sarcoidosis.

## Figures and Tables

**Table 1:** Clinician Demographics and Practice Characteristics.

Characteristics	Category	*n*	%
Sex	Female	77	58.3
	Male	55	41.7
Age	18–30 years	33	25.0
	31–50 years	76	57.6
	51–70 years	23	17.4
Country	Italy	118	89.4
	The Netherlands	8	6.1
	UK	2	1.5
	Belgium	1	0.8
	Denmark	1	0.8
	Germany	1	0.8
	Ireland	1	0.8
Hospital type	University hospital	34	25.8
	General (non-university) teaching hospital	98	74.2

Clinician demographics and practice characteristics of the 132 clinicians provided complete responses. Values are reported as absolute numbers (*n*) and percentages (%).

## Data Availability

Deidentified participant data are available upon reasonable request, subject to review and approval by the local Ethics Committee. Interested researchers may submit a motivated request to the Corresponding Author, who will forward it to the Ethics Committee for evaluation.

## Supplementary Materials

The following supporting information can be downloaded at: https://www.mdpi.com/article/10.3390/jcm14228231/s1, Supplementary Material S1: Clinician Survey Questionnaire.
