# Supplementary material for: Multinational Survey on the Symptom Approach to Diagnosis and Therapy Adopted by Physicians in the Treatment of Gastrointestinal Sarcoidosis
Source: J Clin Med. 2025 Nov 20;14(22):8231. doi: 10.3390/jcm14228231 (PMC12653548; doi:10.3390/jcm14228231)
Supplement: Supplementary file 1 [file jcm-14-08231-s001.zip › jcm-3856212-supplementary.pdf]

Supplementary Material S1 – Clinician Survey Questionnaire

A multinational survey concerning clinician's diagnostic and therapeutic approach to gastrointestinal involvement in sarcoidosis

Introductory welcome screen

Thank you for agreeing to engage in our survey evaluating the diagnostic and therapeutic approach to sarcoidosis-related gastrointestinal (GI) involvement.

Because of its rarity and often non-specific clinical manifestations, an optimal approach to the diagnosis and management of gastrointestinal sarcoidosis is still to be defined.

Your contribution will help to clarify the clinician's approach from a real-life clinical perspective.

The questionnaire should take no longer than 15 minutes (or less) to complete.

Your responses are completely anonymous, and by participating in this survey, you consent to having your responses collected.

We thank you for your time and participation.

CLINICIAN DEMOGRAPHICS

Please select your sex

-Male

-Female

-Other

-Prefer not to specify

How old are you?

<18 years

18-30 years

31-50 years

51-70 years

>70 years

3. In which country do you practice?

■Drop-down selection]

4. What is your primary specialty?

Rheumatology

Pneumology/Pulmonology

Internal (General) Medicine

Gastroenterology

Immunology

General surgery

Gastrointestinal surgery

General practice

Specialist nurse

Advance practitioner

Other (please specify)

5. How long have you been practicing since completing your final professional training experience (e.g., residency or fellowship in medicine)?

0-5 years

6-10 years

11-20 years

21-30 years

>30 years

6. In what type of hospital do you work? (multiple choice allowed)

University hospital

General (non-University Teaching) hospital

General practitioner/family practice clinic

Private practice

-Other (please specify)

7. How many patients do you have under your care with sarcoidosis?

- <25

- 25-30

- 31-50

- 51-100

- >100

8. Approximately how many patients do you have under your care with gastrointestinal complications of sarcoidosis?

- <5 %

- 5-10 %

- 10-30%

- 30-50%

- 50 -70%

- 70 – 90%

- 100%

9. From your perspective, what are the main pathophysiological drivers of GI sarcoidosis? Please select all that apply

Granulomatous infiltration of the mucosa and submucosa layer of GI wall

Granulomatous infiltration of the muscular layer of GI wall

Granulomatous infiltration of all layers of GI wall

CD4/CD8 imbalance

Systemic proinflammatory cytokine activation

Other (please specify)

CLINICAL SURVEY QUESTIONS

A. EVALUATION AND DIAGNOSIS

10. How much do you agree with the following statements?

10a. Clinicians underestimate or do not often recognize sarcoidosis's GI manifestations.

Strongly disagree

Disagree

Neutral

Agree

Strongly agree

10b. Non-specific GI symptoms (e.g., weight loss, epigastric pain, odynophagia, abdominal pain, which may overlap with other conditions) can contribute to delaying the diagnosis of GI sarcoidosis.

Strongly disagree

Disagree

Neutral

Agree

Strongly agree

10c. A multidisciplinary approach to initial evaluation may avoid and/or minimize the diagnostic delay of GI sarcoidosis.

Strongly disagree

Disagree

Neutral

Agree

Strongly agree

11. What are the most common clinical presentations of GI sarcoidosis that you see? Please select your top three choices

Heartburn/reflux

Dysphagia

Unintentional weight loss

Dyspepsia

Distension/bloating

Abdominal pain

Early satiety

Upper gastrointestinal bleeding

Diarrhea

Constipation

12. In the case of suspected GI sarcoidosis, which of the following approaches do you usually favor?

a. Clinical only in the context of an existing sarcoidosis diagnosis

b. Clinical + imaging

c. Clinical + imaging + biopsy

12a. More specifically, which diagnostic tests would you most often order? (please select all that apply)

‘Sarcoid’ type blood tests (e.g., angiotensin convertin enzyme, chitotriosidase)

‘Other’ blood tests (e.g., inflammatory markers)

Faecal testing (e.g., faecal elastase, occult blood)

Functional bowel studies (e.g., barium meal study)

Plain radiography (x-rays)

Computerised tomography (CT)

Magnetic resonance imaging (MRI)

Position emission tomography (PET)

Ultrasound (US)

Endoscopy (gastroscopy, colonscopy)

Surgical biopsy

Others - please specify

13. In your clinical experience, how frequently do you ultimately diagnose GI sarcoidosis involvement by endoscopic biopsy?

<10%

10-30%

31-50%

51-70%

>70%

14. In a patient with existing sarcoidosis and suspected GI involvement, how concerned are you about ruling out other possible differential diagnoses (e.g., inflammatory conditions such as inflammatory bowel diseases or vasculitis, infectious diseases, neurological diseases, neoplastic conditions etc)?

Please rank from 0 to 11

Inflammatory conditions (0-1)

Infectious conditions (0-10)

Malignant conditions (0-1)

Autoimmune conditions (0-1)

Other structural abnormalities (strictures, etc.) (0-11)

15. In the patient(s) with GI sarcoidosis that you have seen, what are the most common coexisting extraintestinal organ systems involved? Rank from 10 (most common) to 0 (least common)

a. Pulmonary involvement

b. Lymphadenopathies

c. Skin lesions

d. Uveitis

e. Parotitis

f. Peripheral arthritis

g. Renal involvement

h. Neurological involvement

i. Splenic involvement

l. Hepatic involvement

m. Pancreatic involvement

n. Cardiac involvement

o. Rhino-laryngeal involvement

16. How often do you consider a differential diagnosis or association between inflammatory bowel diseases (e.g., Crohn's disease, ulcerative colitis) and GI Sarcoidosis?

a. Never

b. Rarely

c. Sometimes

d. Often

e. Always

17. Have you ever experienced “sarcoid-like reactions” in Crohn’s patients treated with Infliximab? If so, has this event led to a re-evaluation of the differential diagnosis between GI sarcoidosis and a drug-induced side effect?

a. Yes, every time it happened, we had a new discussion about the diagnosis.

b. Yes, but we rarely discussed the diagnosis again.

c. Yes, but we have never had a repeat discussion about the diagnosis.

d- No, we have no experienced of this side effect

THERAPY

18. How often do patients with GI sarcoidosis receive care from a gastroenterologist with strong expertise in sarcoidosis?

Never

Rarely

Sometimes

Often

Always

19. How much do you agree with the following statements?

19a. The therapeutic approach to GI sarcoidosis largely depends on which other extraintestinal organ system/s are also involved and/or the existing level of systemic disease control.

Strongly disagree

Disagree

Neutral

Agree

Strongly agree

19b. The therapeutic approach to GI sarcoidosis largely depends on which area(s) of the GI tract is/are involved.

Strongly disagree

Disagree

Neutral

Agree

Strongly agree

20. In your opinion, should glucocorticoid therapy, immunosuppressive and/or biologic therapy be considered as the first-line therapy for symptomatic GI sarcoidosis without other systemic involvement?

Yes

No

Not sure

21. In patients prescribed glucocorticoid therapy for GI sarcoidosis, how often do you co-prescribe GI protective therapy (e.g., PPIs, H2 blockers, PKABs)?

Never

Rarely

Sometimes

Often

Always

23. What is your treatment strategy in patients with GI involvement who do not respond to or tolerate the maximum glucocorticoid dosage? (please select all that apply)

- Continue steroid therapy

- Wean steroid therapy

-Focus on lifestyle, including dietary modifications

-Combination therapy, i.e., continue or reduce current GC dose, and add new drug/s (e.g., immunosuppressive agents such as MTX)

-Substitution, [i.e., stop GC and add new drug/s (e.g., immunosuppressive agents such as MTX)]

-Consider invasive management, including surgical interventions

-Other (please specify)

24. How often do you prescribe corticosteroid-sparing DMARD immunosuppressive therapy (e.g., Methotrexate) for GI involvement in sarcoidosis?

-Never

-Rarely

-Sometimes

-Often

-Always

25. Do you consider biologic therapy (e.g., infliximab, adalimumab, rituximab) as a possible therapeutic alternative in GI sarcoidosis, which is refractory to treatment with glucocorticoids?

-Never

-Rarely

- Sometimes

-Often

-Always

26. What is the expected timeline for clinical response to high-dose glucocorticoid therapy in patients with GI sarcoidosis?

< 1 month

1-2 months

3-5 months

6 months

27. What is the expected timeline for clinical response to DMARD therapy in patients with GI sarcoidosis?

< 1 month

1-2 months

3-5 months

6 months

28. What is the expected timeline for clinical response to biological therapy in patients with GI sarcoidosis?

1-2 months

3-5 months

6 months

29. GI surgery in sarcoidosis should be considered a potential therapeutic strategy, regardless of GC efficacy.

Strongly disagree

Disagree

Neutral

Agree

Strongly agree

30. GI surgery in sarcoidosis may be an effective approach for any involvement of any part of the GI tract (e.g., esophagus, stomach, small and large bowel, ano-rectum)?

30a. Oropharynx

Strongly disagree

Disagree

Neutral

Agree

Strongly agree

30b. Esophagus

Strongly disagree

Disagree

Neutral

Agree

Strongly agree

30c. Stomach

Strongly disagree

Disagree

Neutral

Agree

Strongly agree

30d. Small bowel

Strongly disagree

Disagree

Neutral

Agree

Strongly agree

30e. Large bowel

Strongly disagree

Disagree

Neutral

Agree

Strongly agree

30f. Ano-rectum

Strongly disagree

Disagree

Neutral

Agree

Strongly agree
